# Characterization of *Salmonella* spp. isolated from chickens in Central China

**DOI:** 10.1186/s12917-020-02513-1

**Published:** 2020-08-20

**Authors:** Xin Wang, Honglin Wang, Tingting Li, Feifei Liu, Yiluo Cheng, Xiaodong Guo, Guoyuan Wen, Qingping Luo, Huabin Shao, Zishu Pan, Tengfei Zhang

**Affiliations:** 1 State Key Laboratory of Virology, College of Life Sciences, Wuhan University, Wuhan, 430072 China; 2 Key Laboratory of Prevention and Control Agents for Animal Bacteriosis, Institute of Animal Husbandry and Veterinary, Hubei Academy of Agricultural Sciences, Wuhan, 430064 China; 3 Hubei Animal Disease Prevention and Control Center, Wuhan, 430070 China

**Keywords:** *Salmonella*, Chicken, Antimicrobial resistance, Multilocus sequence typing, Virulence-associated genes

## Abstract

**Background:**

*Salmonella* is an important zoonotic pathogen, and chickens are one of its main hosts. Every year, *Salmonella* infections pose a serious threat to the poultry industry in developing countries, especially China. In this study, a total of 84 *Salmonella* isolates recovered from sick and healthy-looking chickens in central China were characterized by serotyping, MLST-based strain typing, presence of potential virulence factors, and antimicrobial resistance profiles.

**Result:**

Data showed that the main serotypes of *Salmonella* isolates in central China were *Salmonella enterica* serovar Gallinarum biovar Pullorum*, Salmonella enterica* serovar Gallinarum biovar Gallinarum, *Salmonella enterica* serovar Enteritidis and *Salmonella enterica* serovar Typhimurium, and among them, *S*. Pullorum was the dominant type in both sick and healthy-looking chickens, accounting for 43.9 and 46.5%, respectively, while *S*. Enteritidis was only found in healthy-looking chickens. All isolates exhibited higher resistance rates to ampicillin (97.6%), tetracycline (58.3%) and colistin (51.2%), and among these isolates, 49.5% were resistant to more than three drugs in different combinations. *S*. Enteritidis was the most severe multidrug-resistant serotype, which showed higher resistance rates to colistin, meropenem and ciprofloxacin. Multilocus sequence typing (MLST) revealed that *S*. Gallinarum and *S*. Enteritidis isolates were clustered in clade 1, which belonged to two and one STs, respectively. All *S*. Typhimurium isolates were clustered in clade 3, and belonged to three STs. However, *S*. Pullorum were distributed in three clades, which belonged to 7 STs. Twenty-seven virulence-associated genes were detected, and expected *cdtB*, which was absent in all the isolates, the other 26 genes were conserved in the closely related *Salmonella* serogroup D (*S*. Enteritidis, *S*. Pullorum, and *S*. Gallinarum).

**Conclusion:**

*Salmonella* serogroup D was the major subgroup, and *S*. Pullorum was the most common type in sick and healthy-looking chickens in central China. Drug resistance assays showed serious multiple antimicrobial resistances, and *S*. Enteritidis was the most severe drug-resistant serotype. MLST showed that there was correlation between serotypes and genotypes in most *Salmonella* isolates, except *S*. Pullorum, which showed complicated genetic diversity firstly. These results provide important epidemiological information for us to control *Salmonella* in chickens.

## Background

*Salmonella* is an important zoonotic pathogen that causes infectious diseases in animals and humans [1] . Every year, *Salmonella* infection causes not only decreased production performance and even death of poultry, but also contamination of the human food chain, leading to serious economic losses in the poultry industry, as well as being a threat to public health [2]. Although various prevention and control measures, including eradication programs and vaccine and drug use, have been carried out [3], *Salmonella* infection is still one of the most important problems worldwide.

Currently, based on the difference in O, H and Vi antigens, > 2600 different *Salmonella* serotypes have been identified [4]. It is interesting that, although their genomes are similar, the ranges of host in different serotypes are discrepant. *Salmonella enterica* serovar Gallinarum is a host-specific pathogen that only infects birds. It has two biovars, *Salmonella enterica* serovar Gallinarum biovar Gallinarum (*S*. Gallinarum) and *Salmonella enterica* serovar Gallinarum biovar Pullorum (*S*. Pullorum), which cause fowl typhoid and pullorum disease in poultry, respectively [5], leading to great economic losses in the poultry industry, especially in China. *Salmonella enterica* serovar Typhimurium and *Salmonella enterica* serovar Enteritidis are the most common serotypes of *Salmonella*, which can infect broad hosts, including humans and birds. *S*. Typhimurium causes a typhoid-like systemic illness and *S*. Enteritidis is the main cause of acute gastroenteritis in humans [6]. These serotypes of *Salmonella* are considered to be the most important food-borne pathogens worldwide. Other serotypes such as *Salmonella enterica* serovar Heidelberg, *Salmonella enterica* serovar Kentucky and *Salmonella enterica* serovar Newport are also reported in chickens [7]. Because of the differences in pathogenicity and hosts among different serotypes, to understand the dominant serotypes, virulence factors and genetic characteristics of prevailing strains will help us further develop control strategies in the poultry industry.

Antimicrobial resistance and its spread are also a serious problem caused by *Salmonella*. Although the Chinese government is promoting reduction of antibiotic use currently, antimicrobial drugs remain one of the important options for *Salmonella* control [8], which leads to an increase of multiple drug-resistant bacteria worldwide. From 1970 to 2010 in China, the resistance rates to antimicrobials, such as ampicillin, gentamicin, streptomycin, tetracycline, and chloramphenicol, were increasing, and now remain at high levels in *S*. Pullorum [9]. Ampicillin resistance in *Salmonella* has been recognized as the most serious problem in some Asian countries, such as in Bangladesh (68.4%) and Vietnam (80.4%) [10]. The extensive use of antimicrobials in animal production has resulted in contamination by multidrug-resistant *Salmonella* in food animals [11]. Recently, a series of resistance genes for human antimicrobials, such as *mcr-1* and *tetX3/4*, was discovered in zoonotic bacteria [12]. This is a major public health concern because animal-derived antibiotic-resistant bacteria can be transmitted to humans [13].

Poultry is the most important host of *Salmonella*, so assessing the distribution of *Salmonella* in poultry has become important for better prevention and control of its infection. In this study, we investigated the main serotypes of *Salmonella* isolated from sick and healthy-looking chickens, and assessed their genetic relationship among our isolates, presence of potential virulence factors and antimicrobial-resistance profiles.

## Results

### Serotype identification of *Salmonella* isolates from chickens

A total of 84 strains, including 41 from sick chickens and 43 from healthy-looking chickens were identified as *Salmonella*, and further serotyped based on agglutination tests and PCR tests. *Salmonella* serogroup D, including *S*. Enteritidis, *S*. Pullorum and *S*. Gallinarum accounted for 84.76% of our isolates, and among them, *S*. Pullorum was the most common type in both sick chickens (*n* = 18) and healthy-looking chickens (*n* = 20) (Table 1). In addition, 11 isolates (2 from healthy-looking chickens and 9 from sick chickens) were identified as *S*. Typhimurium, and 16 isolates (5 from healthy-looking chickens and 11 from sick chickens) were identified as *S*. Gallinarum. It is interesting that *S*. Enteritidis (*n* = 14) was only found in healthy-looking chickens.

**Table 1:** Numbers and serotypes of *Salmonella* isolated from healthy-looking and sick chickens

Serotypes	*S*. Enteritidis	*S*. Typhimurium	*S*. Gallinarum	*S*. Pullorum	Others	Total
healthy-looking^a^	14	2	5	20	2	43
sick^b^	0	9	11	18	3	41
Total	14	11	16	38	5	84

^a^Healthy-looking chicken; ^b^sick and dead chicken

### Antimicrobial susceptibilities of different serotypes of *Salmonella* isolates

The susceptibility of *Salmonella* isolates to 10 antimicrobials was tested using MIC assays. The isolates showed high resistance rates to ampicillin (97.6%), tetracycline (58.3%), and colistin (51.2%), and lower resistance rates for gentamicin (1.2%), tigecycline (4.8%), ciprofloxacin (6.0%), cefotaxime (7.1%), and meropenem (7.1%) (Table 2). Among these isolates, higher resistance rates to colistin (64.3%), meropenem (35.7%) and ciprofloxacin (28.6%) were observed in *S*. Enteritidis, which can infect humans, while cefotaxime resistance was only observed in *S*. Gallinarum (25%) and *S*. Pullorum (5.3%), which only infect birds. The isolates showed 30 different antimicrobial-resistance patterns, and only one *S*. Gallinarum strain was sensitive to all the antimicrobials tested (Table 3). Forty-nine isolates (59.5%) were resistant to ≥3 antimicrobials in different combinations. Resistance to ampicillin, tetracycline, and colistin was the most common multidrug-resistance profile, accounting for 9.6%. One *S*. Enteritidis isolate was resistant to seven antimicrobials, and six of 14 (42.9%) *S*. Enteritidis isolates were resistant to more than five antimicrobials (Table 3). *S*. Enteritidis showed highest resistance rates among different serotypes of isolates in six of 10 tested antimicrobials, including ampicillin, meropenem, tetracycline, ciprofloxacin, trimethoprim-sulfamethoxazole, and colistin (Table 2).

**Table 2:** No. of isolates in different MICs and antimicrobial resistance of *Salmonella* isolates (*n* = 84)

No. of isolates in different MICs & resistance rates																				
Serotypes	Ampicillin				Cefotaxime				Meropenem				Gentamicin				Tetracycline			
	≤8	16	≥32		≤1	2	≥4		≤1	2	≥4		≤4	8	≥16		≤4	8	≥16	
SE^a^(14)	0	0	14	100.0	14	0	0	0.0	8	1	5	35.7	14	0	0	0.0	0	1	13	92.9
ST^b^(11)	0	0	11	100.0	11	0	0	0.0	5	5	1	9.1	11	0	0	0.0	6	2	3	27.3
SG^c^(16)	2	0	14	87.5	7	5	4	25.0	16	0	0	0.0	13	3	0	0.0	2	1	13	81.3
SGP^d^(38)	0	0	38	100.0	33	3	2	5.3	34	4	0	0.0	35	2	1	2.6	22	0	16	42.1
OT^e^(5)	0	0	5	100.0	4	1	0	0.0	3	2	0	0.0	5	0	0	0.0	1	0	4	80.0
Total(84)			82	97.6			6	7.1			6	7.1			1	1.2			49	58.3
No of isolates in different MICs & resistance rates																				
Subspecies	Tigecycline				Chloramphenicol				Ciprofloxacin				Trimethoprim-sulfamethoxazole				Colistin			
	≤2	4	≥8		≤8	16	≥32		≤1	2	≥4		≤2/38	–	≥4/76		≤2	4	≥8	
SE^a^(14)	14	0	0	0.0	8	1	5	35.7	8	2	4	28.6	7	0	7	50.0	2	3	9	64.3
ST^b^(11)	9	2	0	0.0	6	1	4	36.4	10	0	1	9.1	7	0	4	36.4	4	4	3	27.3
SG^c^(16)	13	2	1	6.3	7	0	9	56.3	16	0	0	0.0	14	0	2	12.5	5	6	5	31.3
SGP^d^(38)	33	2	3	7.9	27	2	9	23.7	35	3	0	0.0	32	0	6	15.8	5	10	23	60.5
OT^e^(5)	5	0	0	0.0	3	0	2	40.0	4	1	0	0.0	4	0	1	20.0	2	0	3	60.0
Total(84)			4	4.8			29	34.5			5	6.0			20	23.8			43	51.2

^a^*S.* Enteritidis; ^b^*S*. Typhimurium; ^c^*S.* Gallinarum; ^d^*S*. Pullorum; ^e^other serotypes;

*Escherichia coli* ATCC 25922 was the quality control, the detailed results are as follows, ampicillin (8 μg/ml), cefotaxime (0.12 μg/ml), meropenem (< 0.125 μg/ml), gentamicin (1 μg/ml), tetracycline (1 μg/ml), tigecycline (0.25 μg/ml), chloramphenicol (4 μg/ml), ciprofloxacin (0.015 μg/ml), trimethoprim-sulfamethoxazole (0.5/9.5 μg/ml) and colistin (2 μg/ml)

**Table 3:** Resistance patterns among the 84 *Salmonella* isolates

Resistance patterns^a^							Prevalence, (n)					
							SE	ST	SG	SGP	OT	total
AMP							0	3	2	7	1	13
TCY							0	0	1	0	0	1
AMP	TCY						1	1	1	5	1	9
AMP	CLS						0	2	0	6	0	8
AMP	TGC						0	0	0	1	0	0
AMP	CHL						0	0	0	1	0	1
AMP	CHL	CLS					0	0	0	2	0	2
AMP	CHL	STX					0	2	0	0	0	2
AMP	MEM	CHL					0	1	0	0	0	1
AMP	MEM	TCY					1	0	0	0	0	1
AMP	TCY	CLS					3	0	0	3	1	7
AMP	TCY	STX					1	1	1	0	0	3
AMP	STX	CLS					0	0	0	4	0	4
AMP	TCY	CHL					0	0	3	1	0	4
AMP	CHL	STX	CLS				1	0	0	0	0	1
AMP	CTX	TCY	CHL				0	0	2	0	0	2
AMP	MEM	TCY	CLS				1	0	0	0	0	1
AMP	TCY	CHL	CLS				0	0	2	3	1	6
AMP	TCY	STX	CLS				0	0	1	1	0	2
AMP	TCY	CLS	TGC				0	0	0	1	0	0
AMP	GEN	STX	CLS	TGC			0	0	0	1	0	1
AMP	CTX	TCY	CHL	CLS			0	0	1	2	0	3
AMP	MEM	TCY	STX	CLS			1	0	0	0	0	1
AMP	TCY	CHL	CIP	STX			2	0	0	0	0	2
AMP	MEM	TCY	CHL	CLS			1	0	0	0	0	1
AMP	TCY	CHL	STX	CLS			0	0	0	0	1	1
AMP	TCY	CIP	STX	CLS			1	0	0	0	0	1
AMP	CTX	TCY	CHL	CLS	TGC		0	0	1	0	0	0
AMP	TCY	CHL	CIP	STX	CLS		0	1	0	0	0	1
AMP	MEM	TCY	CHL	CIP	STX	CLS	1	0	0	0	0	1

^a^Resistance breakpoints were gentamicin (GEN; ≥ 16 μg/mL), meropenem (MEM; ≥ 4 μg/mL), ampicillin (AMP; ≥ 32 μg/mL), cefotaxime (CTX; ≥ 4 μg/mL), colistin (CLS; ≥8 μg/mL), ciprofloxacin (CIP; ≥ 4 μg/mL), chloramphenicol (CHL; ≥ 32 μg/mL), tigecycline (TGC; ≥ 8 μg/mL), compound sulfamethoxazole (SXT; ≥4/76 μg/mL), and tetracycline (TCY; ≥ 16 μg/mL) (Clinical and Laboratory Standards Institute, 2017)

### Genetic diversity of *Salmonella* isolates

**Fig. 1:**
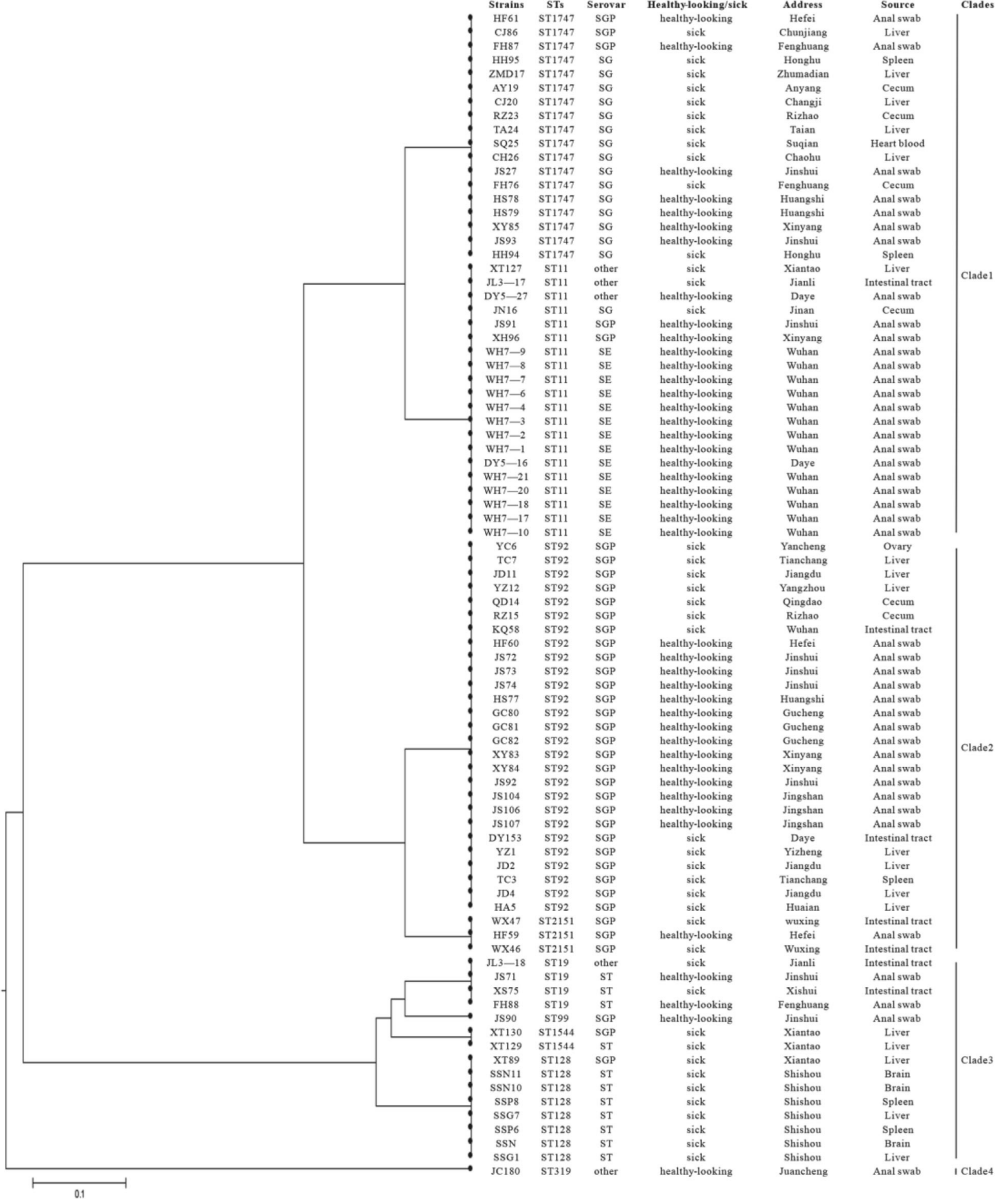
Multilocus sequence typing minimum evolution tree of the *Salmonella* isolates

The *Salmonella* isolates were classified into four clades based on the MLST genotypes (Fig. 1). The allele numbers and the sequences of housekeeping genes in each ST are shown in Additional file 1. All of the *S*. Gallinarum and *S*. Enteritidis isolates were clustered in clade 1, and among them, 15 of 16 *S*. Gallinarum isolates belong to ST-1747, which is not reported currently, while the remaining one *S*. Gallinarum and all the *S*. Enteritidis isolates belonged to ST-11. All the *S*. Typhimurium isolates were clustered in clade 3, and belonged to ST-128, ST-1544 and ST-19. In *S*. Pullorum, 71.1% isolates belonged to the dominant genotype ST-92, while another six ST types were identified, including ST-2151, ST-11, ST-1747, ST128, ST-1544 and ST-99, which showed the genetic diversity of *S*. Pullorum. Among these *S*. Pullorum isolates, three isolates belonged to ST-99, ST-128 and ST-1544 were clustered in clade 3.

### Distribution of virulence-associated genes in different serotypes of *Salmonella* isolates

As shown in Table 4, *cdtB* was not found in all isolates, while the remaining of detected virulence-associated genes were found in all serogroup D *Salmonella* (*S*. Enteritidis, *S*. Pullorum and *S*. Gallinarum). In contrast, *sefA* and *sopE* were absent in one and two *S*. Typhimurium isolates, respectively, and *sodC1* and *sopE* were absent in one other serotype of *Salmonella* (JC180), which was the only isolates in clade 3 in this study.

**Table 4:** Prevalence of virulence-associated genes in avian *Salmonella* spp.

Gene	*S.* Enteritidis	*S.* Typhimurium	*S.* Gallinarum	*S.* Pullorum	Others	Total (%)
	(*n* = 14)	(*n* = 11)	(*n* = 16)	(*n* = 38)	(*n* = 5)	
*sodC1*	•	•	•	•	4	98.8
*spvC*	•	•	•	•	•	100
*spvB*	•	•	•	•	•	100
*spiA*	•	•	•	•	•	100
*pagC*	•	•	•	•	•	100
*cdtB*	°	°	°	°	°	0
*msgA*	•	•	•	•	•	100
*invA*	•	•	•	•	•	100
*sipB*	•	•	•	•	•	100
*prgH*	•	•	•	•	•	100
*spaN*	•	•	•	•	•	100
*orgA*	•	•	•	•	•	100
*tolC*	•	•	•	•	•	100
*iroN*	•	•	•	•	•	100
*sitC*	•	•	•	•	•	100
*lpfC*	•	•	•	•	•	100
*sifA*	•	•	•	•	•	100
*sopB*	•	•	•	•	•	100
*sefA*	•	10	•	•	•	98.8
*pipA*	•	•	•	•	•	100
*ttrC*	•	•	•	•	•	100
*misL*	•	•	•	•	•	100
*siiE*	•	•	•	•	•	100
*mgtB*	•	•	•	•	•	100
*spi4D*	•	•	•	•	•	100
*shdA*	•	•	•	•	•	100
*sopE*	•	9	•	•	4	96.4

• indicates the presence of the gene;

° indicates that the gene does not exist

## Discussion

The distribution of *Salmonella* serovars in poultry differs among countries and regions. In India, *S*. Typhimurium, *S*. Gallinarum and *S*. Enteritidis were the most prevalent serovars, accounting for 96.2% of isolates [14]. In Egypt, *S*. Enteritidis and *S*. Typhimurium were the most commonly identified serotypes recovered from broiler chickens and retail shops [15]. In contrast, an investigation from Japan showed that only *Salmonella enterica* serovar Infantis, *Salmonella enterica* serovar Manhattan and *Salmonella enterica* serovar Schwarzengrund were present in cecal samples of broiler chickens [16], which was different from China. In our study, the main types of isolated *Salmonella* were *S*. Pullorum, *S*. Gallinarum, *S*. Enteritidis, and *S*. Typhimurium, and among them, *S*. Pullorum was the most common type in both healthy-looking and sick chickens. Although eradication programs for *S*. Pullorum from breeding birds are underway in most of the poultry farms, pullorum disease is still one of the most severe diseases in poultry. *S*. Pullorum can cause acute sepsis in chicks, mostly infects young chicks within 20 days of age, and the morbidity and mortality are high [17]. In contrast, carrier state without obvious symptoms frequently occurs in adult chickens, which become an important source of transmission [18]. It is well-known that the transmission of *S*. Pullorum occurs both horizontally and vertically [19], and the strong and multipath transmission might play an important roles in its spread. It is particularly interesting that *S.* Enteritidis was present in healthy-looking chickens, which suggested that the pathogenicity of the prevalent strains was weak in chickens. However, *S*. Enteritidis colonizes the gastrointestinal tract of poultry, resulting in no clinical symptoms in chickens, but causing gastroenteritis in humans [20]. It has become one of the most commonly reported causes of foodborne illness in humans [21]. Although a vaccine is used to control *S.* Enteritidis in the US and Europe [22], there is no good measure for its control in chickens in China. High isolation rate of *S.* Enteritidis during the slaughtering process has been reported in China [23]. We think that carriage in healthy-looking chickens means that there is a high risk of *S.* Enteritidis entering the food processing stage, which highlights its public health threat.

It is thought that animal breeding is an important source of resistant pathogens. Therefore, antimicrobial resistance is a serious problem in the poultry industry as well as a threat to public health. High resistance rates to ampicillin (97.6%), tetracycline (58.3%) and colistin (51.2%) were observed, and 59.5% of isolates were resistant to three or more antimicrobials in different combinations. Therefore, multidrug resistance limits the choices of treatment of *Salmonella* infection in chickens. High resistance rates to ampicillin and tetracycline have been found in isolates from poultry or poultry products in many countries, such as the US (ampicillin, 85%; tetracycline, 35%) [24], Malaysia (ampicillin, 89.5%; tetracycline, 85.1%) [25], and Egypt (ampicillin, 86.7%; tetracycline, 40.0%) [26], as well as in patient with gastroenteritis [27]. However, serious resistance to colistin is seen in only a few countries, including China [28], but colistin used to be considered the last choice for treatment for Gram-negative bacterial infection [29]. What is more important, *S*. Enteritidis which can infect humans, is the most severe drug-resistant serotype. Higher resistance rates to colistin, meropenem and ciprofloxacin, which are often used for human treatment, were observed in this serotype. As mentioned, *S*. Enteritidis is carried in healthy-looking chickens and has a high risk of entering the food chain, so its resistance is closely related to human health. Therefore, this serious problem is not only harmful for poultry industry but also public health.

In this study, the genetic relationship of 84 *Salmonella* isolates was determined by MLST. All of the *S*. Enteritidis and *S*. Gallinarum isolates were clustered in the first subgroup of clade 1, while all the *S*. Typhimurium isolates were clustered in clade 2, suggesting a strong correlation between STs and serotypes. However, *S*. Pullorum isolates spread in clades 1, 2 and 3, which showed complex genetic diversity. Besides previously reported ST-92, which is the dominant ST of *S*. Pullorum, and ST-2151 [30], some STs, including ST-1747, ST-11, ST-99, ST-1544 and ST-128, were firstly identified in *S*. Pullorum isolates. Even three strains (ST-99, ST-1544 and ST-128) showed high genetic similarity to *S*. Typhimurium. As in previous studies, *S*. Gallinarum and *S*. Pullorum, which share the same O antigens 1, 9 and 12, were a direct descendant of *S*. Enteritidis after host adaption [31]. Our results suggested that, there was correlation between serotypes and genotypes in most *Salmonella* isolates. However, *S*. Pullorum has evolved toward diversity with long-term colonization in birds, and this might also be a reason for the high prevalence of *S*. Pullorum among poultry in China.

Although the genetic background of these *Salmonella* serotypes was similar, the host specificity and pathogenicity differed among serotypes. *S*. Typhimurium and *S*. Enteritidis had wide host ranges, and the former caused a typhoid-like systemic illness, while the latter was the main cause of acute gastroenteritis in humans. In contrast, *S*. Pullorum and *S*. Gallinarum were bird specific, and showed different pathological symptoms. We detected the prevalence of 27 virulence-associated genes, which were involved in invasion and intracellular survival, among our isolates. The pathogenicity of *Salmonella* is mainly reflected in its ability to invade non-phagocytic cells, survive in phagocytic cells, and replicate and proliferate in phagocytic cells, all of which are closely related to the virulence factors encoded in the *Salmonella* pathogenicity island (SPI) [32]. For example, inactivation of *sipB* in *Salmonella enterica serotype* Dublin strongly reduces fluid secretion and inflammation [33], and deletion of *prgH* result in strongly reduced virulence of *S.* Typhimurium [34]. Knock out of *sodCI* or *spv* genes reduces persistence of *S*. Enteritidis in mice [35]. We found that these 27 virulence-associated genes were relatively conserved in our *Salmonella* isolates, especially in serogroup D *Salmonella*, and suggested that these there was no direct correlation between serotypes and the distribution of these virulence genes. The genomic sequences of different serotypes revealed that host-restricted *Salmonella* had undergone more extensive degradation than host promiscuous *Salmonella* [36]. According to their study, two of our detected genes, *lpfC* and *shdA*, which were involved in host colonization, were identified as pseudogenes in *S*. Pullorum and *S*. Gallinarum [31]. In addition, removed metabolic pathways due to reduced genomes might be a more important factor for niche adaption processes [31], rather than prevalence of virulence-associated genes. Therefore, we think although these 27 genes were important for the pathogenicity of *Salmonella*, they were not the only key factors for their virulence and host ranges.

## Conclusion

*Salmonella* serogroup D was the major subgroup, and *S*. Pullorum was the most common type in sick and healthy-looking chickens in central China. Drug resistance assays showed high resistance rates to ampicillin, tetracycline and colistin, and among them, *S*. Enteritidis was the most severe drug-resistant serotype, which showed higher resistance rates to colistin, meropenem and ciprofloxacin. MLST showed that there was correlation between serotypes and genotypes in most *Salmonella* isolates, except *S*. Pullorum, which showed complicated genetic diversity. These results provide important epidemiological information for control of *Salmonella* in chickens.

## Methods

### Identification and serotyping of *Salmonella* isolates

During 2013–2018, there were 84 strains of *Salmonella* that were mainly isolated in central China (Henan, Jiangsu and Hubei). Sick chickens were euthanized with an intramuscular injection of sodium pentobarbital (100 mg/kg bodyweight) according to Hubei Province Laboratory Animal Management Regulations – 2005. Samples were isolated from brain, lung, spleen and liver of dead or sick chickens on poultry farms, and isolated from anal swabs of healthy-looking chickens on poultry farms or at poultry markets (Fig. 1). To isolate the *Salmonella*, collected disease samples or anal swabs were directly transported to the laboratory. Each sample was inoculated into 225 mL of buffered peptone water (BPW; Land Bridge, China). After 16 h incubation at 37 °C, 0.1 mL and 10 mL of BPW were transferred into 10 mL of tetrathionate broth and 100 mL of selenite broth, respectively. Subcultures were spread on xylose-lysine-deoxycholate (XLD; Land Bridge, China) agar, xylose-lysine-tergitol 4 (XLT4; Land Bridge, China) agar, and Hektoen (HE; Hopebio, China) agar, and incubated at 37 °C for 24 h. The typical *Salmonella* colonies were confirmed by PCR amplification of the *hut* gene, and the primers were as follows, hut-F, 5′-ATGTTGTCCTGCCCCTGGTAAGAGA-3′, hut-R, 5′-ACTGGCGTTATCCCTTTCTCTGCTG-3′ [37]. Serotypes were determined by a slide agglutination test with O-antigen antiserum and a tube agglutination test with H-antigen antiserum, and confirmed by quadruplex PCR analysis as previously reported [38]. After the experiment, the *Salmonella*-positive chickens were euthanized and underwent harmless treatment based on the regulations from Hubei Provincial Animal Care and Use Committee.

### Antimicrobial resistance profiles

The minimum inhibitory concentrations (MICs) of gentamicin, meropenem, ampicillin, cefotaxime, colistin, tetracycline, ciprofloxacin, chloramphenicol, trimethoprim-sulfamethoxazole and tigecycline were determined according to the Clinical and Laboratory Standards Institute [39]. *Escherichia coli* ATCC 25922 was used for quality control. Isolates were classified as susceptible, intermediate or resistant according to their MICs.

### MLST and phylogenetic tree

To analyze the genetic diversity, MLST was carried out. The isolates were grown overnight in Luria-Bertani medium at 37 °C, and DNA was extracted using MiniBEST Universal Genomic DNA Extraction Kit (TaKaRa, Dalian, China). The following seven housekeeping genes, *aroC*, *dnaN*, *hemD*, *hisD*, *purE*, *sucA*, and *thrA* [40], in each tested isolate were amplified by PCR and the PCR products were sequenced by bi-directional DNA sequencing. The obtained sequences were analyzed using *salmonella* MLST database (http://enterobase.warwick.ac.uk/species/senterica/allele_st_search), and the allele numbers and sequence types (STs) were assigned. The phylogenetic tree was constructed using MEGA cluster analysis.

### Detection of virulence-associated genes

Considering the contribution of virulence genes to the invasiveness and pathogenicity of *Salmonella*, a total of 27 virulence-associated genes were screened by PCR. Twenty seven virulence-associated genes, including *sodC1*, *spvC*, *spvB*, *spiAi, pagC*, *cdtB*, *msgA*, *invA*, *sipB*, *prgH*, *spaN*, *orgA*, *tolC*, *iroN*, *sitC*, *lpfC*, *sifA*, *sopB*, *sefA*, *pipA*, *ttrC*, *misL*, *siiE*, *mgtB*, *spi4D*, *shdA*, and *sopE*, were detected by PCR tests. The primers and amplification conditions were as previously described [41]. PCR was performed in a GeneAmp PCR System 2720 (Applied Biosystems, Singapore). The PCR cycling consisted of 35 cycles of 30 s at 94 °C, 30 s at 55 °C and 1 min at 72 °C and resulting amplification products were separated by electrophoresis in 2% agarose gel, stained with ethidium bromide and visualized under UV light.

## Supplementary information

**Additional file 1.**

## Data Availability

The DNA sequences generated and/or analysed during the current study are available in the GenBank repository, Accession: from MT799111 to MT799173. The others datasets used and/or analyzed during the current study are available from the corresponding author on reasonable request.

## Abbreviations

*S*. Enteritidis: *Salmonella enterica* serovar Enteritidis
*S*. Typhimurium: *Salmonella enterica* serovar Typhimurium
*S*. Gallinarum: *Salmonella enterica* serovar Gallinarum biovar Gallinarum
*S*. Pullorum: *Salmonella enterica* serovar Gallinarum biovar Pullorum
MLST: Multilocus Sequence Typing
STs: Sequence types
MICs: Minimum inhibitory concentrations
